# Uptake of prevention of mother to child transmission interventions in Kenya: health systems are more influential than stigma

**DOI:** 10.1186/1758-2652-14-61

**Published:** 2011-12-28

**Authors:** John Kinuthia, James N Kiarie, Carey Farquhar, Barbra A Richardson, Ruth Nduati, Dorothy Mbori-Ngacha, Grace John-Stewart

**Affiliations:** 1 Department of Obstetrics and Gynaecology, Kenyatta National Hospital/University of Nairobi, Kenya; 2 Department of Global Health, University of Washington, Seattle, WA, USA; 3 Department of Medicine, University of Washington, Seattle, WA, USA; 4 Department of Pediatrics, University of Washington, Seattle, WA, USA; 5 Department of Epidemiology, University of Washington, Seattle, WA, USA; 6 Department of Biostatistics, University of Washington, Seattle, WA, USA; 7 Department of Paediatrics and Child Health, University of Nairobi, Nairobi, Kenya

**Keywords:** mother-to-child HIV transmission, HIV/AIDS, Health system, testing, antiretrovirals, facility delivery

## Abstract

**Background:**

We set out to determine the relative roles of stigma versus health systems in non-uptake of prevention of mother to child transmission (PMTCT) of HIV-1 interventions: we conducted cross-sectional assessment of all consenting mothers accompanying infants for six-week immunizations.

**Methods:**

Between September 2008 and March 2009, mothers at six maternal and child health clinics in Kenya's Nairobi and Nyanza provinces were interviewed regarding PMTCT intervention uptake during recent pregnancy. Stigma was ascertained using a previously published standardized questionnaire and infant HIV-1 status determined by HIV-1 polymerase chain reaction.

**Results:**

Among 2663 mothers, 2453 (92.1%) reported antenatal HIV-1 testing. Untested mothers were more likely to have less than secondary education (85.2% vs. 74.9%, p = 0.001), be from Nyanza (47.1% vs. 32.2%, p < 0.001) and have lower socio-economic status. Among 318 HIV-1-infected mothers, 90% reported use of maternal or infant antiretrovirals. Facility delivery was less common among HIV-1-infected mothers (69% vs. 76%, p = 0.009) and was associated with antiretroviral use (p < 0.001). Although internal or external stigma indicators were reported by between 12% and 59% of women, stigma was not associated with lower HIV-1 testing or infant HIV-1 infection rates; internal stigma was associated with modestly decreased antiretroviral uptake. Health system factors contributed to about 60% of non-testing among mothers who attended antenatal clinics and to missed opportunities in offering antiretrovirals and utilization of facility delivery. Eight percent of six-week-old HIV-1-exposed infants were HIV-1 infected.

**Conclusions:**

Antenatal HIV-1 testing and antiretroviral uptake was high (both more than 90%) and infant HIV-1 infection risk was low, reflecting high PMTCT coverage. Investment in health systems to deliver HIV-1 testing and antiretrovirals can effectively prevent infant HIV-1 infection despite substantial HIV-1 stigma.

## Background

Interventions for the prevention of mother to child transmission (PTMCT) of HIV-1 have the potential to almost eliminate paediatric HIV-1, with effective regimens resulting in a transmission risk of less than 2% [1]. However, the impact of the most effective PMTCT intervention is only as good as population coverage. A highly effective intervention, such as highly active antiretroviral therapy, which can decrease mother to child transmission to about 1%, will be of no benefit to HIV-1-infected women who are not diagnosed with HIV-1.

In 2010, the World Health Organization (WHO) and the Joint United Nations Programme on HIV/AIDS (UNAIDS) estimated that only 56% of HIV-1-infected women accessed PMTCT interventions in Africa, where maternal HIV-1 prevalence is highest [2]. Diagnosis of maternal HIV-1 during pregnancy has been a major bottleneck to delivering interventions in PMTCT programmes. In 2009, it was estimated that in sub-Saharan Africa, HIV-1 testing was available to just over a third of pregnant women, a considerable but still inadequate improvement from 8% reported seven years earlier [3]. Among women offered antenatal HIV-1 testing, acceptance rates of between 55% and 99.8% are reported [4-6]. Women may decline testing because they wish to consult their partners [7-10]. Others may refuse HIV-1 testing following insufficient counselling as they perceive few benefits of testing [11,12]. Opt-out HIV-1 testing overcomes these barriers by routinizing HIV-1 testing within antenatal clinics (ANCs) [13,14].

Following HIV-1 testing, women may not inform their partners of positive HIV-1 test results, fearing stigmatization, abandonment or domestic violence [8,15,16]. Additionally, women remain concerned that their diagnoses will not remain secret [17]. HIV-related discrimination can lead to social isolation. As a result, women may elect to not use ANC services at the site where they received HIV-1 testing, decline facility delivery, or fail to take the antiretrovirals to avoid inadvertent disclosure [18,19].

To maximally decrease paediatric HIV-1 infections, it is essential to assess coverage of services and infant HIV-1 outcomes and to identify barriers to uptake of PMTCT interventions. Barriers may be stigma related or service provision related. It is critical to determine the relative role of each of these potential barriers because the approach to improving programmes would differ based on which is most important. If stigma is the most influential barrier, community efforts to decrease stigma would be critical. Conversely, if systems are more important, focusing on better service delivery would yield effectiveness. In Kenya, more than 90% of mothers take their infants for routine immunizations at six weeks [20]. To determine barriers to uptake of PMTCT interventions, we conducted a study among all mothers bringing their infants for six-week immunizations. This approach to evaluation allowed us to obtain information on mothers who either did or did not access PMTCT services as part of their recent pregnancy care.

## Methods

### Study setting and population

This was a cross-sectional study of all women attending six public sector maternal and child health (MCH) clinics in Kenya for routine infant six-week immunizations: four in Nairobi at Dandora, Mathare North, Babadogo and Kangemi city council clinics, and two in western Kenya at Kisumu and Bondo District hospitals, in Nyanza Province. The MCH clinics evaluated were determined through purposive rather than random sampling. We selected and compared MCH clinics in two provinces with marked differences in HIV-1 prevalence: Nyanza Province had an HIV-1 prevalence of 14.9%, while in Nairobi, the prevalence was 8.8% [21,22].

At the MCH clinics evaluated, HIV-1 testing was routinely offered as part of antenatal care. The clinics additionally provided counselling on infant feeding, antiretroviral drug use and advice on facility delivery as part of routine PMTCT service. The choice of antiretroviral drugs was based on Kenyan Ministry of Health guidelines at the time. Women with CD4 counts of ≤ 350 cells/mm^3 ^or in WHO Stage 3 or 4 were recommended to initiate highly active antiretroviral therapy. The more efficacious short-course zidovudine regimen and or single-dose nevirapine at onset of labour was provided for mothers in WHO Stage 1 or 2 with CD4 counts of > 350 cells/mm^3^. HIV-1-exposed infants were offered HIV DNA PCR testing at the six-week immunization visit.

### Recruitment and data collection

Mother-infant pairs attending the MCH clinic for routine six-week immunizations were recruited. After infant weighing and vaccination, the study nurse explained the study aims and procedures. Following written informed consent, a questionnaire was administered to assess maternal socio-demographic characteristics, stigma indicators, ANC attendance, hospital delivery, and prior participation in PMTCT programmes. Among HIV-1-infected mothers, we additionally inquired on uptake of antiretroviral drugs, infant feeding practices, reasons for non-facility delivery, and non-use of antiretroviral drugs. HIV-1-exposed infants were offered DNA PCR HIV-1 testing.

### Stigma measures

Using standardized questions, which had been previously used in Tanzania, we evaluated four domains of HIV-1 stigma, namely: fear of casual transmission and refusal of contact with people living with HIV/AIDS; value- and morality-related attitudes of blame, judgement and shame for those living with HIV/AIDS; disclosure of HIV test results; and enacted stigma or discrimination [21]. These four domains provide a quantitative measure of HIV-1-related stigma and discrimination [22].

### Data analysis

STATA version 10 (STATA Corp, College Station, Texas, USA) was used to analyze data on testing at antenatal clinics, facility delivery, use of maternal and infant antiretrovirals, and infant feeding. We used Pearson's Chi square and Fisher's exact tests to compare categorical variables, and t tests were used for continuous variables. Multivariate analysis was conducted using logistic regression with those covariates that were significantly (p < 0.05) different on univariate analysis with testing at ANC, antiretroviral drug use and facility delivery in the respective models.

### Ethical approval

Approval for the study was obtained from Human Subjects Division at the University of Washington and the Kenyatta National Hospital Ethical and Research Committee. Authorization was also obtained from Nyanza's Provincial Medical Officer and the Medical Officer of Health, Nairobi.

## Results

### Baseline characteristics

Between September 2008 and March 2009, 2700 mothers were enrolled at the six study sites, 908 (33.6%) and 1792 (66.4%) from sites in Nyanza and Nairobi provinces, respectively. The mean age of mothers was 24 years (95% CI: 23.8-24.2). Most (86.2%) were married, had less than secondary education (75.7%) and were not employed (70.3%). Socio-economic status was assessed by amount paid in monthly rent and ownership of a television set and gas cooker. The mean monthly rent was US$22.6 (95% CI: 21.9-23.2); less than 50% of mothers owned a television set and about 10% owned a gas cooker (Table 1).

**Table 1:** Baseline characteristics of mothers in the cohort

Characteristic	N (%), mean (95% CI)		
**Region**	**Combined** **N = 2700**	**Western** **908 (33.6%)**	**Nairobi** **1792 (66.4%)**

**Socio-demographic and economic**			
Age (years)**	24.0 (23.8-24.2)	23.3 (22.9-23.6)	24.3 (24.1-24.5)
Education lower than secondary level**	2043 (75.7%)	723 (79.6%)	1320 (73.7%)
Married (n = 2662)**	2294 (86.2%)	726 (81.5%)	1568 (88.5%)
Unemployed (n = 2698)**	1897 (70.3%)	598 (66%)	1299 (72.5%)
Economic ($) Monthly rent* (n = 2187)**	22.6 (21.9-23.2)	16.7 (15.2-18.2)	24.5 (21.923.2)
Own television**	1210 (44.8%)	316 (34.8%)	894 (49.9%)
Gas cooker**	267 (9.9%)	56 (6.2%)	211 (11.2%)
			
**Obstetrics**			
Primiparous	987 (36.6%)	321 (35.4%)	666 (37.2%)
		
**Utilization of antenatal and maternity services**			
Attend ANC**	2,638 (97.7%)	865 (95.3%)	1773 (98.9%)
Non-facility delivery (n = 2699)**	743 (27.5%)	340 (37.5%)	403 (22.5%)

SD - standard deviation

*Excludes mothers (especially in rural areas) who own their homes

**p value < 0.05

### HIV-1 testing at Antenatal Clinics

**Figure 1:**
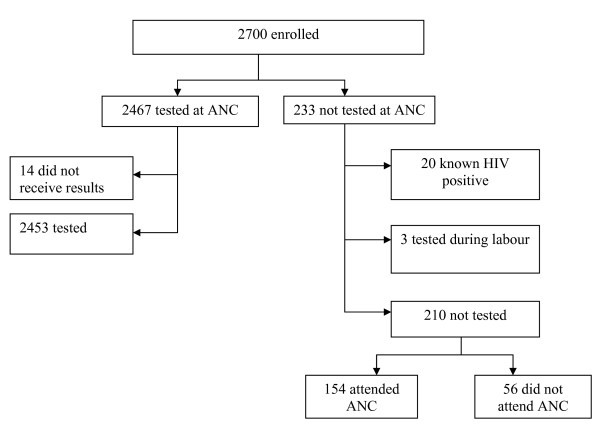
**Antenatal HIV testing among mothers enrolled**.

Of 2700 mothers enrolled in the study, 37 were excluded from the analysis: 14 were tested but did not receive their results, 20 were already known to be HIV-1 infected prior to pregnancy, and three were tested at labour. These mothers were excluded because their HIV-1 status was not identified through the MCH system, which was the focus of this study, or because they did not receive results and would therefore not receive interventions. Overall, 2453 (92.1%) mothers reported having received HIV-1 results during pregnancy (Figure 1).

Compared with mothers who were tested, untested mothers were younger (23.2 vs. 24.0 years, p = 0.039), less educated (85.2% vs. 74.9% had less than secondary education, p = 0.001), less likely to be married (79.5% vs. 86.7%, p = 0.004), and more likely to reside in Nyanza (Table 2). Additionally, mothers not tested were more likely to be of lower socio-economic status as indicated by monthly rent ($18.60 vs. $22.80, p < 0.001), television set ownership (31.9% vs. 46.1%, p < 0.001), or gas cooker ownership (4.8% vs. 10.4%, p = 0.009). No significant differences in employment status or parity were observed between mothers tested and those not tested.

**Table 2:** Comparison of selected characteristics among mothers tested for HIV-1 antenatally and those not tested

	Antenatal HIV testing (n = 2663)		
**Characteristics**	**Not tested** **% mean** **(95% CI)** **210 (7.9%)**	**Tested** **% mean** **(95% CI)** **2453 (92.1%)**	**P value**

**Socio-demographic and obstetrics**			
Age	23.2 (5.4)	24.0 (5.0)	0.039
Education lower than secondary level	179 (85.2%)	1836 (74.9%)	0.001
Married (n = 2629)	163 (79.5%)	2102 (86.7%)	0.004
Unemployed (n = 2661)	156 (74.3%)	1712 (69.9%)	0.177
Primiparous	73 (34.8%)	905 (36.9%)	0.539
Home delivery (n = 2662)	114 (54.3%)	620 (25.3%)	< 0.001
**Economic**			
Monthly rent in US$ (n = 2159)	18.6 (12.5)	22.8 (14.8)	< 0.001
Own TV (n = 2663)	67 (31.9%)	1131 (46.1%)	< 0.001
Gas cooker (n = 2663)	10 (4.8%)	255 (10.4%)	0.009
**Stigma measures** **Fear of casual transmission (n = 2660)**			
Not buy food from PLHIV* not visibly sick	49 (23.4%)	502 (20.5%)	0.310
Not buy food from PLHIV* visibly sick	110 (52.6%)	1125 (45.9%)	0.061
**Internal stigma**			
HIV is punishment for bad behaviour (n = 2582)	115 (56.9%)	1225 (51.5%)	0.136
People with HIV should be ashamed (n = 2608)	25 (12.2%)	336 (14.0%)	0.477
**Enacted sigma**			
Abandoned by spouse/partner (n = 2512)	96 (49.0%)	1142 (49.3%)	0.929
**Disclosure**			
Disclosed HIV status to partner	2169 (94%)	-	
**Region (n = 2663)**			
Nyanza (western Kenya)	99 (47.1%)	789 (32.2%)	< 0.001

* PLHIV - people/person living with HIV/AIDS

The proportion of mothers who would not buy food from a person living with HIV/AIDS who was not visibly sick did not differ significantly between tested and untested mothers. However, there was a trend for mothers who were not tested to not want to buy food from a vendor who was visibly sick, although the amount of difference was modest (52.6% vs. 45.9%, p = 0.06). Responses to measures of internal and enacted stigma and rates of disclosure of HIV-1 status to partner did not differ between tested and untested mothers. In multivariate analysis, higher education (OR = 1.72, 95% CI: 1.14-2.60, p = 0.009), socio-economic status (as measured by television set ownership) (OR = 1.43, 95% CI: 1.02-1.96, p = 0.03) and Nairobi Province (OR = 1.69, 95% CI: 1.26-2.27, p < 0.001) were found to be independently associated with having been HIV-1 tested.

Of the 210 mothers not tested for HIV-1, 154 (73%) were not tested despite having attended antenatal clinics (ANCs); the remaining 56 had not attended antenatal care clinics. Reasons why mothers did not attend ANCs included competing time demands (30.4%), distance (19.6%), cost (16.1%), lack of perceived need (19.6%), to avoid HIV-1 testing (7.1%), being in school (7.1%), social problems (3.6%), and attitudes of facility staff (3.6%). Reasons why mothers who attended ANCs were not tested included unavailability of services or failure of staff to offer testing (43.5%) and slow service provision (12.3%). Personal factors included need to consult partner (13.6%), fear of results (12.3%), no perceived need due to previous negative test (7.8%), and cost when mothers visited private antenatal clinics (3.2%).

### Utilization of PMTCT interventions by HIV-1-infected women

#### Facility delivery

In total, 336 (13.7%) mothers were diagnosed antenatally with HIV-1. HIV-1-positive mothers were more likely to have a non-facility delivery than HIV-1-uninfected mothers (31.0% vs. 24.3% p = 0.009) (Table 3). Among HIV-1-infected mothers, those who did not deliver at a health facility were less educated (91.4% vs. 75.9% had less than secondary education, p = 0.001) and were of lower socio-economic status. Age, marital status, parity and distance to the health facility did not differ between HIV-1-infected women who delivered at a facility and those who did not. There was a trend for mothers who did not deliver in a health facility to report that people with HIV-1 should be ashamed. However, responses to other stigma measures were comparable between mothers who delivered at a health facility and those who did not (Table 2). In multivariate analysis, higher education (OR = 2.9, 95% CI: 1.3-6.1, p = 0.007) and socio-economic status (as measured by television set ownership) (OR = 2.1, 95% CI: 1.2-3.7, p = 0.006) were independently associated with facility delivery in HIV-1-infected mothers.

**Table 3:** Correlates of facility delivery and use of antiretroviral drugs

	Delivery (n = 336)			ARV drug use (n = 318)		
	Non Facility N % Mean (95% CI) 104 (31%)	Facility N % Mean (95% CI) 232 (69.1%)	P value	Did not use N % Mean (95% CI) 31 (9.8%)	Used N % Mean (95% CI) 287 (90.3%)	P value
**Socio-demographic characteristics**						
Age	26.3 (25.2-27.4)	25.7 (25-26.3)	0.363	25.3 (23.6-27)	26 (25.3-26.6)	0.440
Lower than secondary education	95 (91.4%)	176 (75.9%)	0.001	24 (77.4%)	230 (80.1%)	0.720
Married	84 (86.6%)	194 (87.8%)	0.769	23 (79.3%)	240 (88.6%)	0.150
Unemployed	70 (67.3%)	154 (66.4%)	0.867	20 (64.5%)	191 (66.6%)	0.820
Primiparous	17 (16.4%)	59 (25.4%)	0.066	5 (16.1%)	67 (23.3%)	0.362
Home delivery	-	-	-	18 (58.1%)	75 (26.1%)	< 0.001
Distance to health facility	2.9 (2-3.8)	2.8 (2.3-3.3)	0.82	-	-	-
**Economic**						
Monthly rent in US$ (n = 248)	13.0 (11.1-14.9)	23.7(21-26.3)	< 0.001	14.2 (11-17.4)	22(19.7-24.3)	< 0.001
Own TV	23 (22.1%)	95 (41.0%)	0.001	8 (25.8%)	106 (36.9%)	0.202
Gas cooker	2 (1.9%)	21 (9.1%)	0.018*	0 (0%)	23 (8.0%)	0.102
**Stigma measures** **Fear of casual transmission**						
Not buy food from PLHIV not visibly sick	12 (11.5%)	15 (6.5%)	0.114	2 (6.5%)	23 (8.0%)	> 0.999*
Not buy food from PLHIV visibly sick	28 (26.9%)	56 (24.1%)	0.586	10 (32.3%)	69 (24.0%)	0.315
**Internal stigma**						
HIV is punishment for bad behaviour	44 (46.3%)	82 (38.0%)	0.167	17 (58.6%)	101 (37.8%)	0.030
People with HIV should be ashamed	12 (11.7%)	13 (5.7%)	0.056	7 (22.6%)	15 (5.3%)	< 0.001
**Enacted sigma**						
Abandoned by spouse/partner	47 (48.0%)	123 (54.2%)	0.302	13 (46.4%)	148 (53.1%)	0.504
**Disclosure**						
Disclosed HIV status to partner	72 (81.8%)	159 (81.1%)	0.889	20 (87.0%)	196 (80.0%)	0.584
**Region**						
Nyanza (western Kenya)	56 (53.9%)	112 (48.3%)	0.345	15 (48.4%)	142 (49.5%)	0.908

* Fischer's exact test

The most common reason given for non-facility delivery was security concern when labour started at night (25%). Other reasons given were distance (23.1%), cost (17.3%), rapid progression of labour (17.3%), being alone at home (3.8%) or failure to secure a vehicle (2.9%). Only 5% of mothers stated a preference to be delivered by a traditional birth attendant.

#### Antiretroviral use

Overall, 90% of HIV-1-infected mothers used PMTCT antiretrovirals (ARVs) or gave infants ARVs. Maternal ARVs were not dispensed to 10.7% of mothers, and of those given drugs, 10.6% failed to take them. Among infants, 15.7% were not dispensed ARVs, but only 2.2% were not given if the drugs were dispensed. The 10% of mothers who did not use maternal or infant ARVs were more likely to have had a non-facility delivery (58.1% vs. 26.1%, p < 0.001) (Table 3). In addition, they were more likely to report they thought HIV-1 was a punishment for bad behaviour (58.6% vs. 37.8%, p = 0.030) and that people with HIV-1 should be ashamed (22.6% vs. 5.3%, p < 0.001) (Table 3). In multivariate analysis, non-facility delivery (OR = 0.28, 95% CI: 0.13-0.60, p = 0.001) and thinking that people with HIV-1 should be ashamed (OR = 0.22, 95% CI: 0.08-0.62, p = 0.004) were independently associated with failure to use ARVs.

#### Infant feeding

Most (92.4%) HIV-1-infected mothers were feeding their infants as recommended by WHO (exclusively breastfeeding, replacement feeding, or wet nursing). The majority (77.2%) reported exclusively breastfeeding. Mothers who had disclosed their HIV-1 status to their partners were more likely to feed their infants as recommended (82.3% vs. 57.9%, p = 0.01).

#### Infant HIV-1 transmission

Of 336 HIV-1-exposed infants, HIV-1 DNA PCR results were available for 300. Of these, seven (2.3%) had indeterminate results, 270 (90.0%) were HIV-1 uninfected and 23 (7.7%) were HIV-1 infected. Fifteen HIV-1 exposed infants were not tested, 10 (66.7%) because they had a specimen for the test taken at another facility. Mothers who did not use antiretrovirals were more likely to transmit HIV-1 to their infants (9.4% vs. 6.7%, p = 0.5). However, we were not adequately powered to show a statistically significant difference. Level of education, marital status, employment status, place of delivery, parity and measures of the four domains of HIV-1 stigma did not differ between mothers of HIV-1-infected and uninfected infants.

## Discussion

In Africa, where about 90% of the world's new paediatric HIV-1 infections occur, attaining high PMTCT coverage has the potential to contribute substantially to eradicating infant HIV-1 infection globally. In this study of PMTCT delivery in Nairobi and western Kenya, we found remarkably high coverage of PMTCT services in six sampled public sector sites, with 92% of women receiving antenatal HIV-1 testing, 90% of whom received maternal or infant antiretrovirals. In contrast to low global estimates of PMTCT coverage, the coverage we found reflects expansion of PMTCT services in Kenya and demonstrates much higher levels of ANC testing and PMTCT services than in previous years; this is consistent with promising recent reports from other sub-Saharan African settings. As other settings seek to improve PMTCT coverage, our study results are encouraging and suggest that United Nations General Assembly goals of 80% coverage are widely attainable.

While noting efficacy of the system, we identified areas for further improvement. Of 2700 women enrolled, 210 (7.9%) mothers were not tested for HIV-1, missing an opportunity to access PMTCT interventions. Most of these mothers had attended ANCs, surmounting one of the traditional obstacles to PMTCT access. The most common reason cited by mothers for not testing was unavailability of HIV-1 testing services at the ANC or failure of providers to offer testing. In public facilities, this was possibly due to stock outs of test kits. Private facilities may not have offered HIV-1 testing due to concerns that mothers would avoid facilities that conduct HIV-1 testing. Another disincentive for HIV-1 testing was time required for counselling and/or testing services. Combined, these health-system based factors contributed to about 60% of non-testing among mothers who attended antenatal clinics.

Four domains of HIV-1 stigma were evaluated using questions that had been tested and validated in Tanzania [21,22]. These domains were: fear of casual transmission and refusal of contact with people living with HIV/AIDS; value- and morality-related attitudes of blame, judgement and shame for those living with HIV/AIDS; disclosure of HIV test results; and enacted stigma or discrimination. Surprisingly, although stigma was prevalent, stigma measures did not differ significantly between women who were HIV-1 tested and those who were not, suggesting a diminishing role of stigma as a barrier to HIV-1 testing at ANC clinics. This may be due to successful "opt-out" approaches, which seek to routinize and de-stigmatize HIV-1 testing [13,14]. We found that mothers with lower education, from Nyanza Province and those of lower socio-economic status were less likely to have been tested. Women of lower socio-economic status face constraints, including transport costs to access services. We found the most common reason given by mothers for failure to attend antenatal clinics was competing time demands.

HIV-1 testing in pregnancy is ultimately futile in preventing infant HIV-1 if mothers do not use PMTCT interventions. In this study, 336 mothers were identified antenatally as HIV-1 infected. Of these, 104 (31%) did not deliver at a health facility. Facility delivery offers opportunity to prevent prolonged labour and rupture of membranes, both contributing factors to transmission of HIV-1 [23,24]. Additionally, facility delivery facilitates use of ARVs. Although the proportion of non-facility delivery we recorded was lower than the 56% reported by the Kenya Demographic Health Survey in the general population [20,25], it was of concern that HIV-1-infected mothers were significantly less likely to deliver at facilities than HIV-1-uninfected mothers. Most impediments cited by mothers for not delivering at facilities could be overcome if programmes incorporated birth preparedness interventions [26]. During antenatal care, PMTCT providers should assist mothers to plan for birth, educate mothers to recognize signs of labour, and help them plan for transport to the facility [26].

In 2009, global estimates suggested that almost half of pregnant HIV-1-infected women in sub-Saharan Africa did not receive ARVs [2]. Home delivery, low levels of education, being unmarried, non-disclosure of test results, few antenatal care visits, and poor interactions with healthcare providers have been reported as barriers to use antiretrovirals for PMTCT [27-31]. In our study, although 90% of mother-infant pairs received maternal *or *infant ARVs, 20% of mothers and 17% of infants did not use ARVs.

We identified two barriers to use of ARVs. First, about 10% of mothers and 15% of infants were not given ARVs despite attending antenatal clinics and being identified as HIV-1 infected. Second, mothers who did not deliver at a health facility were less likely to use ARVs, similar to reports in other studies [27,28,32,33]. When mothers received ARVs, self-reported adherence was high. Almost 90% of mothers reported using maternal dose and 97.7% reported giving the drugs to their infants, comparable with other studies [28,29]. Among the 10% of mothers who did not take maternal or infant ARVs, internal stigma indicators were significantly higher, suggesting that stigma may have compromised this minority of women. Determining strategies to preserve confidentiality while delivering ARVs in this group of women may be useful to increase uptake beyond 90%.

Most mothers in our study reported exclusively breastfeeding as recommended by WHO [34]. Exclusive breastfeeding confers a lower risk of HIV-1 transmission than mixed feeding [35]. The 2008 Kenya Demographic Health Survey reported that only 35% of infants younger than three months exclusively breastfed [20]. Encouragingly, we found prevalence of mixed feeding at six weeks was 7.6%. This suggests that messages on exclusive breastfeeding are being translated into practice. We found that mothers who had not disclosed their HIV-1 status to their partners were more likely to mixed feed, underscoring the importance of facilitating disclosure of HIV-1 status to partners.

The HIV-1 transmission risk in this study was 7.7%. This included women who did not attend antenatal care and is considerably lower than expected without PMTCT interventions (about 20%). Overall, it reflects high coverage and uptake of PMTCT at these sites and as more comprehensive PMTCT interventions are implemented, the infant HIV-1 transmission risk would be expected to decrease further. Infant HIV-1 infection risk was not associated with socio-demographic or stigma variables, suggesting that if systems are built, women will come for testing and infants will be spared HIV-1 infection despite a variety of potentially daunting social constraints. Our findings are consistent with data from a study in KwaZulu-Natal, South Africa, that similarly tested infants brought for immunization at six weeks of age [36]. Both studies offer objective evaluation of programme effectiveness.

This study had several strengths. Through targeting mothers who brought their infants for immunizations at six weeks, we were able to access both mothers who had utilized antenatal care and those who did not. At six weeks postpartum, HIV-1 testing, delivery, ARV utilization and choice of infant feeding would all have been implemented. Therefore, it was possible to assess utilization of PMTCT interventions during antenatal, intrapartum and postpartum periods and to include assessment of infant HIV-1 as an outcome of programme success. Importantly, in contrast with previous studies, we systematically ascertained stigma indicators to probe whether these were associated with intervention uptake and with infant HIV-1.

Limitations of the study included purposive rather than random selection of clinics, which theoretically limits generalizability. The study was designed with an estimate that 10% of mothers would not have attended antenatal clinics [25]; however, we found that only 2.3% of mothers had not received antenatal care. One benefit of the unexpectedly high ANC attendance in our study was that it allowed us to comprehensively probe barriers to PMTCT delivery within the system. However, the study did not assess mothers who did not bring their children for immunizations and who may have also been less likely to have accessed PMTCT.

In summary, the PMTCT coverage we observed in routine national programmes was encouragingly high. There were missed opportunities in HIV-1 testing, ARVs and facility delivery that modestly lowered the effectiveness of the PMTCT programme. Overall, health systems factors rather than stigma were barriers to utilization of PMTCT services. With more effective PMTCT regimens, as recommended in the recent WHO guidelines, even lower infant HIV-1 risk would be expected in the future. Despite considerable perceived stigma, women access and comply with PMTCT interventions. Thus, efficient PMTCT service delivery has the potential to rapidly and substantially diminish the paediatric HIV-1 epidemic in Africa.

## Competing interests

The authors declare that they have no competing interests.

## Authors' contributions

JK undertook conception and design of the study, data acquisition, analysis, interpretation and drafting of the manuscript. JNK assisted with study design, implementation, analysis and manuscript drafting. CF assisted with data interpretation and manuscript drafting. BR assisted with statistical analyses and manuscript writing. RN assisted with study design, data interpretation and manuscript drafting. DMN assisted with study design and data interpretation. GJS provided primary mentorship of JK, oversight of study conception and design, data analysis, manuscript writing and obtained grant funding. All authors read and approved the final manuscript.
